# Supplementary material for: Heat adaptation of phage T7 under an extended genetic code
Source: Virus Evol. 2021 Dec 1;7(2):veab100. doi: 10.1093/ve/veab100 (PMC8923235; doi:10.1093/ve/veab100)
Supplement: veab100_Supp [file veab100_supp.zip › Sequencing_Analysis.zip!/Sequencing_Analysis/S2/evidence/SNP_11.html]

BRESEQ :: Evidence


| Predicted mutation | | | | | | |
| --- | --- | --- | --- | --- | --- | --- |
| evidence | seq id | position | mutation | annotation | gene | description |
| RA | NC\_001604.1 | 29,304 | A→C | intergenic (–/–) | – / – | –/– |

| Read alignment evidence... | | | | | | | | | | | |
| --- | --- | --- | --- | --- | --- | --- | --- | --- | --- | --- | --- |
|  | seq id | position | | ref | new | freq | score (cons/poly) | reads | annotation | genes | product |
| \* | NC\_001604.1 | 29,304 | 0 | A | C | 98.3% | 14673.4 / 15.5 | 4809 | intergenic (–/–) | *–/–* | –/– |
| Reads supporting (aligned to +/- strand):  **ref** base A (35/45);  **new** base C (2316/2388);  **total** (2361/2448) | | | | | | | | | | | |
| Fisher's exact test for biased strand distribution *p*-value = 3.68e-01 | | | | | | | | | | | |
| Kolmogorov-Smirnov test that lower quality scores support variant *p*-value = 9.95e-01 | | | | | | | | | | | |

Only 100 of 4838 total aligned reads displayed.  

`CGGTCTGGTTACTGGCGCAATCCCATCTGATGCTCAAGCCACACAGCTTATAAGCCAAGCGTTCAGTGACGCTTCTAGCCGTGCTGGTGGTGCTGACTTCCTGATGCGAGTCGGTGACAAGAAGGTAACACTTAACGGAGCCACTACGACTTACCGAGAGTTGATTGGTGAGGAACAGTGGAACGCTCTCATGGTCACAGCACAACGTTCTCAGTTTGAGACTGACGCGAAGCTGAACGAGCAGTATCGCTTGAAGATTAACTCTGCGCTGAACCAAGAGGACCCAAGGACAGCTTGGGAGATGCTTCAAGGTATCAAGGCTGAACTAGATAAGGTCCAACCTGATGAGCAGATGACACCACAACGTGAGTGGCTAATCTCCGCACAGGAACAAGTTCAGAATCAGATGAACGCATGGACGAAAGCTCAGGCCAAGGCTCTGGACGATTCCATGAAGTCAATGAACAAACTTGACGTAATCGACAAGCAATTCCAGAAGCGAATCAACGGTGAGTGGGTCTCAACGGATTTTAAGGATATGCCAGTCAACGAGAACACTGGTGAGTTCAAGCATAGCGATATGGTTAACTACGCCAATAA  >  NC_001604.1/29005‑29604`  

                                                                                                                                                                                                                                                                                                           `|`                                                                                                                                                                                                                                                                                                              
`cGGTCTGGTTACTGGCGCAATCCAATCTGATGCTCAAGCCACACAGCTTATAAGCCAAGCGTTCAGTGACGCTTCTAGCCGTGCTGGTGGTGCTGACTTCCTGATGCGAGTCGGTGACAAGAAGGTAACACTTAACGGAGCCACTACGACTTACCGAGAGTTGATTGGTGAGGAACAGTGGAACGCTCTCATGGTCACAGCACAACGTTCTCAGTTTGAGACTGACGCGAAGCTGAACGAGCAGTATCGCTTGGAGATTAACTCTGCGCTGAACCAAGAGGACCCAAGGACAGCTTGGgag                                                                                                                                                                                                                                                                                                             <  1:221505/301‑1 (MQ=255)`  
    `cTGGTTACTGGCGCAATCCCATCTGATGCTCAAGCCACACAGCTTATAAGCCAAGCGTTCAGTGACGCTTCTAGGCGTGCTGGTGGTGCTGACTTCCTGATGCGAGTCGGTGACAAGAAGGTAACACTTAACGGAGCCACTACGACTTACCGAGAGTTGATTGGTGAGGAACAGTGGAACGCTCTCATGGTCACAGCACAACGTTCTCAGTTTGAGACTGACGCGAAGCTGAACGAGCAGTATCGCTTGGAGATTAACTCTGCGCTGAACCAAGAGGACCCAAGGACAGCTTGGGCGATGc                                                                                                                                                                                                                                                                                                         <  1:692128/301‑1 (MQ=255)`  
    `cTGGTTACTGGCGCAATCCCATCTGATGCTCAAGCCACACAGCTTATAAGCCAAGCGTTCAGTGACGCTTCTAGCCGTGCTGGTGGTGCTGACTTCCTGATGCGAGTCGGTGACAAGAAGGTAACACTTAACGGAGCCACTACGACTTACCGAGAGTTGATTGGTGAGGAACGGTGGAACGCTCTCATGGTCACAGCACAACGTTCTCAGTTTGAGACTGACGCGAAGCTGAACGAGCAGTATCGCTTGGAGATTAACTCTGCGCTGAACCAAGAGGACCCAAGGACAGCTTGGGCGATGc                                                                                                                                                                                                                                                                                                         <  1:825274/301‑1 (MQ=255)`  
         `aaCTGGCGCAATCCCAGCGGATGCTCAAGCCACACAGCTTATAAGCAAAGCGTTCAGTGACGCTGCTAGCCGTGCTGGTGGTGCTGACTTCCTGATGCGAGTCGGAGACAAGAAGGTAACACTTAACGGAGCCACTACGACTTACCGAGAGTTGATTGGTGAGGAACAGTGGAACGCTCTCATGGTCACAGCACAACGTTCTCAGTTTGAGACTGACGCGAAGCTGAACGAGCAGTATCGCTTGGAGATTAACTCTGCGCTGAACCAAGAGGACCCAAGGACAGCTTGGGCGATGCTTCaa                                                                                                                                                                                                                                                                                                    <  1:336341/300‑1 (MQ=255)`  
           `cTGGCGCAATCCCATCTGATGCTCAAGCCACACAGCTTATAAGCCAAGCGTTCAGTGACGCTTCTAGCCGTGCTGGTGGTGCTGACTTCCTGATGCGAGTCGGTGACAAGAAGGTAACACTTAACGGAGCCACTACGACTTACCGAGAGTTGATTGGTGAGGAACAGTGGAACGCTCTCATGGTCACAGCACAACGTTCTCAGTTTGAGACTGACGCGAAGCTGAACGAGCAGTATCGTTTGGAGATTAACTCTGCGCTGAACCAAGAGGACCCAAGGACAGCTTGGGCGATGCTTCAAgg                                                                                                                                                                                                                                                                                                  <  1:409213/301‑1 (MQ=255)`  
             `ggCGCAATCCCATCTGATGCTCAAGCCACACAGCTTATAAGCCAAGCGTTCAGTGACGCTTCTAGCCGTGCTGGTGGTGCTGACTTCCTGATGCGAGTCGGTGACAAGAAGGTAACACTTAACGGAGCCACTACGACTTACCGAGAGTTGATTGGTGAGGAACAGTGGAACGCTCTCATGGTCACAGCACAACGTTCTCAGTTTGAGACTGACGCGAAGCTGAACGAGCAGTATCGCTTGGAGATTAACTCTGCGCTGAACCAAGAGGACCCAAGGACAGCTTGGGCGATGCTTCAAGGTa                                                                                                                                                                                                                                                                                                >  1:676416/1‑301 (MQ=255)`  
                   `aTCCCATCTGATGCTCAAGCCACACAGCTTATAAGCCAAGCGTTCAGTGACGCTTCTAGCCGTGCTGGTGGTGCTGACTTCCTGATGCGAGTCGGTGACAAGAAGGTAACACTTAACGGAGCCACTACGACTTACCGAGAGTTGATTGGTGAGGAACAGTGGAACGCTCTCATGGTCACAGCACAACGTTCTCAGTTTGAGACTGACGCGAAGCTGAACGAGCAGTATCGCTTGGAGATTAACTCTGCGCTGAACCAAGAGGACCCAAGGACAGCTTGGGCGATGCTTCAAGGTATCAAgg                                                                                                                                                                                                                                                                                          >  1:610972/1‑301 (MQ=255)`  
                   `aTCCCATCTGATGCTCAAGCCACACAGCTTATAAGCCAAGCGTTCAGTGACGCTTCTAGCCGTGCTGGTGGTGCTGACTTCCTGATGCGAGTCGGTGACAAGAAGGTAACACTTAACGGAGCCACTACGACTTACCGAGAGTTGATTGGTGAGGAACAGTGGAACGCTCTCATGGTCACAGCACAACGTTCTCAGTTTGAGACTGACGCGAAGCTGAACGAGCAGTATCGCTTGGAGATTAACTCTGCGCTGAACCAAGAGGACCCAAGGACAGCTTGGGCGATGCTTCAAGGTATCAAgg                                                                                                                                                                                                                                                                                          >  1:657364/1‑301 (MQ=255)`  
                     `cccATCTGATGCTCAAGCCACACAGCTTATAAGCCAAGCGTTCAGTGACGCTTCTAGCCGTGCTGGTGGTGCTGACTTCCTGATGCGAGTCGGTGACAAGAAGGTAACACTTAACGGAGCCACTACGACTTACCGAGAGTTGATTGGTGAGGAACAGTGGAACGCTCTCATGGTCACAGCACAACGTTCTCAGTTTGAGACTGACGCGAAGCTGAACGAGCAGTATCGCTTGGAGATTAACTCTGCGCTTAACCAGGAGGACCCAAGGACAGCTTGGGCGATGCTTCAAggctgtctcttt                                                                                                                                                                                                                                                                                        >  1:190988/1‑291 (MQ=255)`  
                     `cccATCTGATGCTCAAGCCACACAGCTTATAAGCCAAGCGTTCAGTGACGCTTCTAGCCGTGCTGGTGGTGCTGACTTCCTGATGCGAGTCGGTGACAAGAAGGTAACACTTAACGGAGCCACTACGACTTACCGAGAGTTGATTGGTGAGGAACAGTGGAACGCTCTCATGGTCACAGCACAACGTTCTCAGTTTGAGACTGACGCGAAGCTGAACGAGCAGTATCGCTTGGAGATTAACTCTGCGCTGAACCAAGAGGAACCAAGGACAGCTTGGGCGATGCTTCCAGGTATCAAGGCt                                                                                                                                                                                                                                                                                        >  1:550488/1‑301 (MQ=255)`  
                     `cccATCTGATGCTCAAGCCACACAGCTTATAAGCCAAGCGTTCAGTGACGCTTCTAGCCGTGCTGGTGGTGCTGACTTCCTGATGCGAGTCGGTGACAAGAAGGTAACACTTAACGGAGCCACTACGACTTACCGAGAGTTGATTGGTGAGGAACAGAGGAACGCTCTCATGGTCACAGCACAACGTTCTCAGTTTGAGACTGACGCGAAGCTGAACGAGCAGTATCGCTTGGAGATTAATTCTGCGCTGAACCAAGAGGACCCAAGGACAGCTTGGGCGATGCTTCAAGGTATCAAGGCt                                                                                                                                                                                                                                                                                        >  1:711895/1‑301 (MQ=255)`  
                       `cATCTGATGCTCAAGCCACACAGCTTATAAGCCAAGCGTTCAGTGACGCTTCTAGCCGTGCTGGTGGTGCTGACTTCCTGATGCGAGTCGGTGACAAGAAGGTAACACTTAACGGAGCCACTACGACTTACCGAGAGTTGATTGGTGAGGAACAGTGGAACGCTCTCATGGTCACAGCACAACGTTCTCAGTTTGAGACTGACGCGAAGCTGAACGAGCAGTATCGCTTGGAGATTAACTCTGCGCTGAACCAAGAGGACCCAAGGACAGCTTGGGCGATGCTTCAAGGTATTAAGGCTGa                                                                                                                                                                                                                                                                                      >  1:775168/1‑301 (MQ=255)`  
                              `gtgtataagagacaggCTTATAAGCCAAGCGTTCAGTGACGCTTCTAGCCGTGCTGGTGGTGCTGACTTCCTGATGCGAGTCGGTGACAAGAAGGTAACACTTAACGGAGCCACTACGACTTACCGAGAGTTGATTGGTGAGGAACAGTGGAACGCTCTCATGGTCACAGCACAACGTTCTCAGTTTGAGACTGACGCGAAGCTGAACGAGCAGTATCGCTTGGAGATTAACTCTGCGCTGAACCAATAGGACCCAAGGACAGCTTGGGCGATGCTTCAAGGTATCAAGGCTGAACTAGAt                                                                                                                                                                                                                                                                               <  1:154110/286‑1 (MQ=255)`  
                              `agCTAAAGCCAAATAGCTTATAAGAAAAGCGTTCAGTGACGCTTCTAGCCGTGCTGGTGGTGCTGACTTCCTGATGCGAGTCGGTGACAAGAAGGTAACACTTAACGGAGCCACTACGACTTACCGAGAGTTGATTGGTGAGGAACAGTGGAACGCTCTCATGGTCACAGCACAACGTTCTCAGTTTGAGACTGACGCGAAGCTGAACGAGCAGTATCGCTTGGAGATTAACTCTGCGCTGAACCAAGAGGACCCAAGGACAGCTTGGGCGATGCTTCAAGGTATCAAGGCTGAACTAGAt                                                                                                                                                                                                                                                                               <  1:559447/300‑1 (MQ=255)`  
                                  `cAAGCCACACAGCTTATAAGCCAAGCGTTCAGTGACGCTTCTAGCCGTGCTGGTGGTGCTGACTTCCTGATGCGAGTCGGTGACAAGAAGGTAACACTTAACGGAGCCACTACGACTTACCGAGAGTTGATTGGTGAGGAACAGTTGAACTCTCTCATGGTCACAGCACAACGTTCTCAGTTTGAGACTGACGCGAAGCTGAACGAGCAGTATCGCTTGGAGATTAACTCTGCGCTGAACCAAGAGGACCCAAGGACAGCTTGGGCGATGCTTCAAGGTATCAAGGCTGAACTAGATAAgg                                                                                                                                                                                                                                                                           <  1:817175/301‑1 (MQ=255)`  
                                  `cAAGCCACACAGCTTATAAGCCAAGCGTTCAGTGACGCTTCTAGCCGTGCTGGTGGTGCTGACTTCCTGATGCGAGTCGGTGACAAGAAGGTAACACTTAACGGAGCCACTACGACTTACCGAGAGTTGATTGGTGAGGAACAGTGGAACGCTCTCATGGTCACAGCACAACGTTCTCAGTTTGAGACTGACGCGAAGCTGAACGAGCAGTATCGCTTGGAGATTAACTCTGCGCTGAACCAAGAGGACCCAAGGACAGCTTGGGCGATGCTTCAAGGTATCAAGGCTGAACTAGATAAgg                                                                                                                                                                                                                                                                           >  1:64668/1‑301 (MQ=255)`  
                                   `aaGCCACAGAAGTTATAAGGAAAGCGTGCAGTGAAGCTTCTAGGCGTGCTGGTGGGTCTGACTTCCTGATGCGAGTCGGTGACAAGAAGGTAACACTTAACGGAGCCACTACGACTTACCGAGAGTTGATTGGTGAGGAACAGTGGAACGCTCTCATGGTCACAGCACAACGTTCTCAGTTTGAGACTGACGCGAAGCTGAACGAGCAGTATCGCTTGGAGATTAACTCTGCGCTGAACCAAGAGGACCCAAGGACAGCTTGGGCGATGCTTCAAGGTATCAAGGCTGAACTAGATAAGGt                                                                                                                                                                                                                                                                          <  1:401644/301‑1 (MQ=255)`  
                                         `cacaGCTTATAAGCCAAGCGTTCAGTGACGCTTCTAGCCGTGCTGGTGGTGCTGACTTCCTGATGCGAGTCGGTGACAAGAAGGTAACACTTAACGGAGCCACTACGACTTACCGAGAGTTGATTGGTGAGGAACAGTGGAACGCTCTCATGGTCACAGCACAACGTTCTCAGTTTGAGACTGACGCGAAGCTGAACGAGCAGTATCGCTTGGAGATTAACTCTGCGCTGAACCAAGAGGACCCAAGGACAGCTTGGGCGATGCTTCAAGGTATCAAGGCTGAACTAGATAAGGTCCAAcc                                                                                                                                                                                                                                                                    <  1:597022/301‑1 (MQ=255)`  
                                         `cacaGCGTATAAGCCAAGCGTTCAGCGACGCTTCTAGCCGTGCTGGTGGTGCTGACTTCCTGATGCGAGTCGGTGACAAGAAGGTAACACTTAACGGAGCCACTACGACTTACCGAGAGTTGATTGGTGAGGAACAGTGGAACGCTCTCATGGTCACAGCACAACGTTCTCAGTTTGAGACTGACGCGAAGCTGAACGAGCAGTATCGCTTGGAGATTAACTCTGCGCTGAACCAAGAGGACCCAAGGACAGCTTGGGCGATGCTTCAAGGTATCAAGGCTGAACTAGATAAGGTCCAAcc                                                                                                                                                                                                                                                                    <  1:261108/301‑1 (MQ=255)`  
                                         `aacaGCTTATAAGCCAAGCGTTCAGTGACGCTTCTAGCCGTGCTGGTGGTGCTGACTTCCTGATGCGAGTCGGTGACAAGAAGGTAACACTTAACGGAGCCACTACGACTTACCGAGAGTTGATTGGTGAGGAACAGTGGAACGCTCTCATGGTCACAGCACAACGTTCTCAGTTTGAGACTGACGCGAAGCTGAACGAGCAGTATCGCTTGGAGATTAACTCTGCGCTGAACCAAGAGGACCCAAGGACAGCTTGGGCGATGCTTCAAGGTATCAAGGCTGAACTAGATAAGGTCCAAcc                                                                                                                                                                                                                                                                    <  1:749844/300‑1 (MQ=255)`  
                                             `gCTTATAAGCCAAGCGTTCAGTGACGCTTCTAGCCGTGCTGGTGGTGCTGACTTCCTGATGCGAGTCGGTGTCAAGATGGTAACACTTAACTGCGCCACTTCTACTTACCGAGATTTTTTTGGTTAGTTCCAGTGGAACGCTCTCTTTGTCACAGCACTACGTTCTCAGTTTGAGACTGACGCCAATCTTAACGAGCAGTATCGCTTGGAGATTAACTCTGCTTTTTTCCCTGAGGACCCAAGTACATCTTGGTCGATGCTTCAAGGTATCAATGCTGAACTAGATAAGGTCCAAcccttt                                                                                                                                                                                                                                                                >  1:589222/1‑297 (MQ=255)`  
                                              `cTTATAAGCCAAGCGTTCAGTGACGCTTCTAGCCGTGCTGGTGGTGCTGACTTCCTGATGCGAGTCGGTGACAAGAAGGTAACACTTAACGGAGCCACTACGACTTACCGAGAGTTGATTGGTGAGGAACAGTGGAACGCTCTCATGGTCACAGCACAACGTTCTCAGTTTGAGACTGACGCGAAGCTGAACGAGCAGTATCGCTTGGAGATTAACTCTGCGCTGAACCAAGAGGACCCAAGGACAGCTTGGGCGATGCTTCAAGGTATCAAGGCTGAACTAGATAAGGTCCAACCtgatg                                                                                                                                                                                                                                                               <  1:380560/301‑1 (MQ=255)`  
                                                 `ataAGCCAAGCGTTCAGGGACGCTTCTAGCCGTGCTGGTGGTGCTGACTTCCTGATGCGAGTCGGTGACAAGAAGGTAACACTTAACGGAGCCACTACGACTTACCGAGAGTTGATTGGTGAGGAACAGTGGAACGCTCTCATGGTCACAGCACAACGTTCTCAGTTTGAGACTGACGCGAAGCTGAACGAGCAGTATCGCTTGGAGATTAACTCTGCGCTGAACCAAGAGGACCCAAGGACAGCTTGGGCGATGCTTCAAGGTATCAAGGCTGAACTAGATAAGGTCCAACCTGATGAGc                                                                                                                                                                                                                                                            <  1:734084/301‑1 (MQ=255)`  
                                                          `gtgtgcAGTGACGCTTCTAGACGGGCGGGTGGTGCTGACTTCCTGATTCGAGTCGGTGACAAGAAGGTAACACTTAACGGAGCCACTACGACTTACCGAGAGTTGATTGGTGAGGAACAGTGGAACGCTATCATGGTCACAGCACAACGTTCTCAGTTTGAGACTGACGCGAAGCTGAACGAGCAGTATCGCTTGGAGATTAACTCTGCGCTGAACCAAGAGGACCCAAGGACAGCTTGGGCGATGCTTCAAGGTATCAAGGCTGAACTAGATAAGGTCCAACCTGATGAGCAGATGacac                                                                                                                                                                                                                                                   <  1:253689/296‑1 (MQ=255)`  
                                                          `gCGTTCAGTGACGCTTCTAGCCGTGCTGGTGGTGCTGACTTCCTGATGCGAGTCGGTGACAAGAAGGTAACACTTAACGGAGCCACTACGACTTACCGAGCGTTGATTGGTGAGGAACAGTGGAACGCTCTCATGGTCACAGCACAACGTTCTCAGTTTGAGACTGACGCGAAGCTGAACGAGCAGTATCGCTTGGAGATTAACTCTGCGCTGAACCAAGAGGACCCAAGGACAGCTTGGGCGATGCTTCAAGGTATCAAGGCTGAACTAGATAAGGTCCAACCTGATGAGCAGATGacac                                                                                                                                                                                                                                                   >  1:29493/1‑301 (MQ=255)`  
                                                            `gTTCAGTGACGCTTCTAGCCGTGCTGGTGGTGCTGACTTCCTGATGCGAGTCGGTGACAAGAAGGTAACACTTAACGGAGCCACTACGACTTACCGAGAGTTGATTGGTGAGGAACAGTGGAACGCTCTCATGGTCACAGCACAACGTTCTCAGTTTGAGACTGACGCGAAGCTGAACGAGCAGTATCGCTTGGAGATTAACTCTGCGCTGAACCAAGAGGACCCAAGGACAGCTTGGGCGATGCTTCAAGGTATCAAGGCTGAACTAGATAAGGTCCAACCTGATGAGCAGATGAcacca                                                                                                                                                                                                                                                 >  1:758129/1‑301 (MQ=255)`  
                                                                 `gTGACGCTTCTAGCCGTGCTGGTGGTGCTGACTTCCTGATGCGAGTCGGTGACAAGAAGGTAACACTTAACGGAGCCACTACGACTTACCGAGAGTTGATTGGTGAGGAACAGTGGAACGCTCTCATGGTCACAGCACAACGTTCTCAGTTTGAGACTGACGCGAAGCTGAACGAGCAGTATCGCTTGGAGATTAACTCTGCGCTGAACCAAGAGGACCCAAGGACAGCTTGGGCGATGCTTCAAGGTATCAAGGCTGAACTAGATAAGGTCCAACCTGATGAGCAGATGACACCACAACg                                                                                                                                                                                                                                            >  1:36524/1‑301 (MQ=255)`  
                                                                 `gTGACGCTTCTAGCCGTGCTGGTGGTGCTGACTTCCTGATGCGAGACGGTGACAAGAAGGTAACACTTAACGGAGCCACTACGACTTACCGAGAGTTGATTGGTGAGGAACAGTGGAACGCTCTCATGGTCACAGCACAACGTTCTCAGTTTGAGACTGACGCGAAGCTGAACGAGCAGTATCGCTTGGAGATTAACTCTGCGCTGAACCAAGAGGACCCAAGGACAGCTTGGGCGATGCTTCAAGGTATCAAGGCTGAACTAGATAAGGTCCAACCTGATGAGCAGATGACACCACAACg                                                                                                                                                                                                                                            <  1:705760/301‑1 (MQ=255)`  
                                                                       `cTTCTAGCCGTGCTGGTGGTGCTGACTTCCTGATGCGAGTCGGTGACAAGAAGGTAACACTTAACGGAGCCACTACGACTTACCGAGAGTTGATTGGTGAGGAACAGTGGAACGCTCTCATGGTCACAGCACAACGTTCTCAGTTTGAGACTGACGCGAAGCTGAACGAGCAGTATCGCTTGGAGATTAACTCTGCGCTGAACCAAGAGGACCCAAGGACAGCTTGGGCGATGCTTCAAGGTATCAAGGCTGAACTAGATAAGGTCCAACCTGATGAGCAGATGACACCACAACGTAAGTg                                                                                                                                                                                                                                      >  1:500165/1‑301 (MQ=255)`  
                                                                                `gTGCTGGTGGTGCTGACTTCCTGATGCGAGTCGGTGACAAGAAGGTAACACTTAACGGAGCCACTACGAATTACCGAGAGTTGATTGGTGAGGAACAGTGGAACGCTCTCATGGTCACAGCACAACGTTCTCAGTTTGAGACTGACGCGAAGCTGAACGAGCAGTATCGCTTGGAGATTAACTCTGCGCTGAACCAAGAGGACCCAAGGACAGCTTGGGCGATGCTTCAAGGTATCAAGGCTGAACTAGATAAGGTCCAACCTGATGAGCAGATGACACCACAACGTGAGTGGCTAAtctc                                                                                                                                                                                                                             <  1:575923/301‑1 (MQ=255)`  
                                                                                            `cTGACTTCCTGATGCGAGTCGGTGACAAGAAGGTAACACTTAACGGAGCCACTACGACTTACCGAGAGTTGATTGGTGAGGAACAGTGGAACGCTCTCATGGTCACAGCACAACGTTCTCAGTTTGAGACTGACGCGAAGCTGAACGAGCAGTATCGCTTGGAGATTAACTCTGCGCTGAACCAAGAGGACCCAAGGACAGCTTGGGCGATGCTTCAAGGTATCAAGGCTGAACTAGATAAGGTCCAACCTGATGAGCAGATGACACCACAACGTGAGTGGCTAATCTCCGCACAGGAACa                                                                                                                                                                                                                 <  1:279571/301‑1 (MQ=255)`  
                                                                                                 `ttCCTGATGCGAGTCGGTGACAAGAAGGTAACACTTAACGAAGCCACTACGACTTACCGAGAGTTGATTGGTGAGGAACAGTGGAACGCTCTCATGGTCACAGCACAACGTTCTCAGTTTGAGACTGACGCGAAGCTGAACGAGCAGTATCGCTTGGAGATTAACTCTGCGCTGAACCAAGAGGACCCAAGGACAGCTTGGGCGATGCTTCAAGGTATCAAGGCTGAACTAGATAAGGTCCAACCTGATGAGCAGATGACACCACAACGTGAGTGGCTAATCTCCGCACAGGAACAAGTTc                                                                                                                                                                                                            <  1:529333/301‑1 (MQ=255)`  
                                                                                                   `ccTGATGCGAGTCGGTGACAAGAAGGTAACACTTAACGGAGCCACTACGACTTACCGAGAGTTGATTGGTGAGGAACAGTGGAACGCTCTCATGGTCACAGCACAACGTTCTCAGTTTGAGACTGACGCGAAGCTGAACGAGCAGTATCGCTTGGAGATTAACTCTGCGCTGAACCAAGAGGACCCAAGGACAGCTTGGGCGATGCTTCAAGGTATCAAGGCTGAACTAGATAAGGTCAAACCTGATGAGCAGATGACACCACAACGTGAGTGGCTAATCTCCGcacctgtctcttataca                                                                                                                                                                                                          >  1:168499/1‑287 (MQ=255)`  
                                                                                                      `ggagatgtgtataagagacagggTAACACTTAACGGAGCCACTACGACTTACCGAGAGTTGATTGGTGAGGAACAGTGTAACGCTCTCATGGTCACAGCACAACGTTCTCAGTTTGAGACTGACGCGAAGCTGAACGAGCAGTATCGCTTGGAGATTAACTCTGCGCTGAACCAATAGGACCCAAGGACAGCTTGGGCGATGCTTCAAGGTATCAAGGCTGAACTAGATAAGGTCCAACCTGATGAGCAGATGACACCACAACGTGAGTGGCTAATCTCCGCACAGGAACAAGTTCAGAAt                                                                                                                                                                                                       <  1:343596/280‑1 (MQ=255)`  
                                                                                                                       `agaagGTAACACTTAACGGAGCCACTACGACTTACCGAGAGTTGATTGGTGAGGAACAGTGGAACGCTCTCATGGTCACAGCACAACGTTCTCAGTTTGAGACTGACGCGAAGCTGAACGAGCAGTATCGCTTGGAGATTAACTCTGCGCTGAACCAAGAGGACCCAAGGACAGCTTGGGCGATGCTTCAAGGTATCAAGGCTGAACTAGATAAGGTCCAACCTGATGAGCAGATGACACCACAACGAGAGTGGCTAATCTCCGCACAGGAACAAGTTCAGAATCAGATGAACGCATGGag                                                                                                                                                                                      >  1:307531/1‑300 (MQ=255)`  
                                                                                                                           `ggTAACACTTAACGGAGCCACTACGACTTACCGAGAGTTGATTGGTGAGGAACAGTGGAACGCTCTCATGGTCACAGCACAACGTTCTCAGTTTGAGACTGACGCGAAGCTGAACGAGCAGTATCGCTTGGAGATTAACTCTGCGCTGAACCAAGAGGACCCAAGGACAGCTTGGGCGATGCTTCAAGGTATCAAGGCTGAACTAGATAAGGTCCAACCTGATGAGCAGATGACACCACAACGTGAGTGGCTAATCTCCGCACAGGAACAAGTTCAGAATCAGATGAACGCATGGACGaaa                                                                                                                                                                                  >  1:782597/1‑301 (MQ=255)`  
                                                                                                                            `ttAACACTTAACGGAGGCACTACGACTTACCGAGAGTTGAGTGGTGAGGAACAGTGGAACGCTGTCATGGTCACAGCAAAACGTTCTCAGTTTGAGACTGACGCGAAGCTGAACGAGCAGTATCGCTTGTAGATTAACGCTGCGCTGAGCCAAGAGGACCCAAGGACAGCTTGGGCGATGCTTCAAGGTATCAAGGCTGAACTAGATAAGGTCCAACCTGATGAGCAGATGACACCACAACGTGAGTGGCTAATCTCCGCACAGGAACAAGTTCAGAATCAGATGAACGCATGGACGAAAg                                                                                                                                                                                 <  1:632554/300‑1 (MQ=255)`  
                                                                                                                                `cacTTAACGGAGCCACTACGACTTACCGAGAGTTGATTGGTGAGGAACAGTGGAACGCTCTCATGGTCACAGCACAACGTTCTCAGTTTGAGACTGACGCGAAGCTGAACGAGCAGTATCGCTTGGAGATTAACTCTGCGCTGAACCAAGAGGACCCAAGGACAGCTTGGGCGATGCTTCAAGGTATCAAGGCTGAACTAGATAAGGTCCAACCTGATGAGCAGATGACACCACAACGTGAGTGGCTAATCTCCGCACAGGAACAAGTTCAGAATCAGATGAACGCATGGACGAAAGCTCa                                                                                                                                                                             >  1:538698/1‑301 (MQ=255)`  
                                                                                                                                           `gCCACTACGACTTACCGAGAGTTGATTGGGGAGGAACAGTGGAACGCTCTCATGGTCACAGCACAACGTTCTCAGTTTGAGACTGACACGAAGCTGAACGAGCAGTATCGCTTGGAGATTAACTCTGCGCTGAACCAAGAGGACCCAAGGACAGCTTGGGCGATGCTTCAAGGTATCAAGGCTGAACTAGATAAGGTCCAACCTGATGAGCAGATGACACCACAACGTGAGTGGCTAATCTCCGCACAGGAACAAGTTCAGAATCAGATGAACGCATGGACGAAAGCTCAGGCCAAGGctc                                                                                                                                                                  <  1:520781/301‑1 (MQ=255)`  
                                                                                                                                             `cACTACTACTTACCGAGAGTTGATTGGTGAGGAACAGTGGAACGCTCTCATGGTCACAGCACAACGTTCTCAGTTTGAGACTGACGCGAAGCTGAACGAGCAGTATCGCTTGGAGATTAACTCTGCGCTGAACCAAGAGGACCCAAGGACAGCTTGGGCGATGCTTCAAGGTATCAAGGCTGAACTAGATAAGGTCCAACCTGATGAGCAGATGACACCACAACGTGAGTGGCTAATCTCCGCACAGGAACAAGTTCAGAATCAGATGAACGCATGGACGAAAGCTCAGGCCAAGGCCCTg                                                                                                                                                                >  1:145020/1‑301 (MQ=255)`  
                                                                                                                                                `tACGACTTACCGAGAGTTGATTGGTGAGGAACAGTGGAACGCTCTCATGGTCACAGCACAACGTTCTCAGTTTGAGACTGACGCGAAGCTGAACGAGCAGTATCGCTTGGAGATTAACTCTGCGCTGAACCAAGAGGACCCAAGGACAGCTTGGGCGATGCTTCAAGGTATCAAGGCTGAACTAGATAAGGTCCAACCTGATGAGCAGATGACACCACAACGTGAGTGGCTAATCTCCGCACAGGAACAAGTTCAGAATCAGATGAACGCATGGACGAAAGCTCAGGCCAAGGCTCTGGAc                                                                                                                                                             <  1:359109/301‑1 (MQ=255)`  
                                                                                                                                                `tACGACTTACCGAGAGTTGATTGGTGAGGAACAGTGGAACGCTCTCATGGTCACAGCACAACGTTCTCAGTTTGAGACTGACGCGAAGCTGAACGAGCAGTATCGCTTGGAGATTAACTCTGCGCTGAACCAAGAGGACCCAAGGACAGCTTGGGCGATGCTTCAAGGTATCAAGGCTGAACTAGATAAGGTCCAACCTGATGAGCAGATGACACCACAACGTGAGTGGCTAATCTCCGCACAGGAACAAGTTCAGAATCAGATGAACGCATGGACGAAAGCTCAGGCCAAGGATCTGGAc                                                                                                                                                             <  1:669608/301‑1 (MQ=255)`  
                                                                                                                                                `tACGACTTACCGAGAGTTGATTGGTGAGGAACAATGGAACGCTCTCATGGTCACAGCACAACGTTCTCAGTTTGAGACTGACGCGAAGCTGAACGAGCAGTATCGCTTGGAGATTAACTCTGCGCTGAACCAAGAGGACCCAAGGACAGCTTGGGCGATGCTTCAAGGTATCAAGGCTGAACTAGATAAGGTCCAACCTGATGAGCAGATGACACCACAACGTGAGTGGCTAATCTCCGCACAGGAACAAGTTCAGAATCAGATGAACGCATGGACGAAAGCTCAGGCCAAGGCTCTGGAc                                                                                                                                                             <  1:161066/301‑1 (MQ=255)`  
                                                                                                                                                `aaCGACTGACCGAGAGGTGATTGGTGAGGAAAAGTGGAACGCTCTCATGGTCACAGCACAACGGTCTCAGTTTGAGACTGACGCGAAGCTGAACGAGCAGTATCGCTTGGAGATTAACGCTGCGCTGAACCAAGAGGACCCAAGGACAGCGTGGGCGATGCTTCAAGGTATCAAGGCTGAACTAGATAAGGTCCAACCTGATGAGCAGATGACACCACAACGTGAGTGGCTAATCTCCGCACAGGAACAAGTTCAGAATCAGATGAACGCATGGACGAAAGCTCAGGCCAAGGCTCTGGAc                                                                                                                                                             <  1:204497/300‑1 (MQ=255)`  
                                                                                                                                                     `attACCGAGAGTTGATGGGTGAGGAACAGTGGAACGCTCTCATGGTCACAGCACAACGTTCTCAGTTTGAGACTGACGCGAAGCTGAACGAGCAGTATCGCTTGGAGATTAACTATGCGCTGAATCAAGAGGACCCAAGGACAGCTTGGGCGATGCTTCAAGGTATCAAGGCTGAACTAGATAAGGTCCAACCTGATGAGCAGATGACACCACAACGTGAGTGGCTAATCTCCGCACAGGAACAAGTTCAGAATCAGATGAACGCATGGACGAAAGCTCAGGCCAAGGCTCTGGACGATTc                                                                                                                                                        <  1:432583/300‑1 (MQ=255)`  
                                                                                                                                                        `aCCGAGAGTTGATTGGTGAGGAACAGTGGAACGCTCTCATGGTCACAGCACAACGTTCTCAGTTTGAGACTGACGCGAAGCTGAACGAGCAGTATCGCTTGGAGATTAACTCTGCGCTGAACCAAGAGGACCCAAGTACAGCTTGGGCGATGCTTCAAGGTATCAAGGCTGAACTAGATAAGGTCCAACCTGATGAGCAGATGACACCACAACGTGAGTGGCTAATCTCCGCACAGGAACAAGTTCAGAATTAGATGAACGCATGGACGAACGCTCAGGCCAAGGCTCTGGACGATTCCAt                                                                                                                                                     >  1:122643/1‑301 (MQ=255)`  
                                                                                                                                                        `aCCGAGAGTTGATTGGTGAGGAACAGTGGAACGCTCTCATGGTCACAGCACAACGTTCTCAGTTTGAGACTGACGCGAAGCTGAACGAGCAGTATCGCTTGGAGATTAACTCTGCGCTGAACCAAGAGGACCCAAGGACAGCTTGGGCGATGCTTCAAGGTATCAAGGCTGAACTAGATAAGGTCCAACCTGATGAGCAGATGACACCACAACGTGAGTGGCTAATCTCCGCACAGGAACAAGTTCAGAATCAGATGAACGCATGGACGAAAGCTCAGGCCAAGGCTCTGGAcctgtctct                                                                                                                                                     >  1:423613/1‑293 (MQ=255)`  
                                                                                                                                                         `ccGAGAGTTGATTGGTGAGGAACAGTGGAACGCTCTCATGGTCACAGCACAACGTTCTCAGTTTGAGACTGACGCGAAGCTGAACGAGCAGTATCGCTTGGAGATTAACTCTGCGCTGAACCAAGAGGACCCAAGGACAGCTTGGGCGATGCTTCAAGGTATCAAGGCTGAACTAGATAAGGTCCAACCTGATGAGCAGATGACACCACAACGTGAGTGGCTAATCTCCGCACAGGAACAAGTTCAGAATCAGATGAACGCATGGACGAAAGCTCAGGCCAAGGCTCTGGCCGATTCCATg                                                                                                                                                    >  1:809592/1‑301 (MQ=255)`  
                                                                                                                                                          `gacaggTTGATTGGTGAGGAACAGTGGAACGCTCTCATGGTCATAGCACAACGTTCTCAGTTTGAGACTGACGCGAAGCTGAACGAGCAGTATCGCTTGGAGATTAACTCTGCGCTGAACCAAGAGGACCCAAGGACAGCTTGGGCGATGCTTCAAGGTATCAAGGCTGAACTAGATAAGGTCCAACCTGATGAGCAGATGACACCACAACGTGAGTGGCTAATCTCCGCACAGGAACAAGTTCAGAATCAGATGAACGCATGGACGAAAGCTCAGGCCAAGGCTCTGGACGATTCCATGa                                                                                                                                                   <  1:625574/296‑1 (MQ=255)`  
                                                                                                                                                          `cGAGAGTTGATTGGTGAGGAACAGGGGAACGCTCTCATGGTCACAGCACAACGTTCTCAGTTTGAGACTGACGCGAAGCTGAACGAGCAGTATCGCTTGGAGATTAACTCTGCGCTGAACCAAGAGGACCCAAGGACAGCTTGGGAGATGCTTCAAGGTATCAAGGCTGAACTAGACAAGGTCCAACCTGATGAGCAGATGACACCACAACGTGAGTGGCTAATCTCCGCACAGGAACAAGTTCAGAATCAGATGAACGCATGGACGAAAGCTCAGGCCAAGGCTCTGGACGATTCCATGa                                                                                                                                                   <  1:313546/301‑1 (MQ=255)`  
                                                                                                                                                             `gagTTGATTGGTGAGGAACAGTGGAACGCTCTCATGGTCACAGCACAACGTTCTCAGTTTGAGACTGACGCGAAGCTGAACGAGCAGTATCGCTTGGAGATTAACTCTGCGCTGAACCAAGAGGACCCAAGGACAGCTTGGGCGATGCTTCAAGGTATCAAGGCTGAACTAGATAAGGTCCAACCTGATGAGCAGATGACACCACAACGTGAGTGGCTAATCTCCGCACAGGAACAAGTTCAGAATCAGATGAACGCATGGACGAAAGCCCAGGCCAAGGCTCTGGACGATTCCATGAAGt                                                                                                                                                >  1:246160/1‑301 (MQ=255)`  
                                                                                                                                                               `gTTGATTGGTGAGGAACAGTGGAACGCTCTCATGGTCACAGCACAACGTTCTCAGTTTGAGACTGACGCGAAGCTGAACGAGCAGTATCGCTTGGAGATTAACTCTGCGCTGAACCAAGAGGACCCAAGGACAGCTTGGGCGATGCTTCAAGGTATCAAAGCTGAACTAGATAAGGTCCAACCTGATGAGCAGATGACACCACAACGTGAGTGGCTAATCTCCGCACAGGAACAAGTTCAGAATCAGATGAACGCATGGACGAAAGCTCAGGCCAAGGCTCTGGAAGATTCCATGAAGTCa                                                                                                                                              >  1:286515/1‑301 (MQ=255)`  
                                                                                                                                                                     `gagacaGGAACAGTGGAACGCTCTCATGGTCACAGCACAACGTTCTCAGTTTGAGACTGACGCGAAGCTGAACGAGCAGTATCGCTTGGAGATTAACTCTGCGCTGAACCAAGAGGACCCAAGGACAGCTTGGGCGATGCTTCAAGGTATCAAGGCTGAACTAGATAAGGTCCAACCTGATGAGCAGATGACACCACAACGTGAGTGGCTAATCTCCGCACAGGAACAAGTTCAGAATCAGATGAACGCATGGACGAGAGCTCAGGCCAAGGCTCTGGACGATTCCATGAAGTCAATGAAc                                                                                                                                        <  1:604926/296‑1 (MQ=255)`  
                                                                                                                                                                      `ggTGAGGAACAGTGGAACGCTCTCATGGTCACAGCACAACGTTCTCAGTTTGAGACTGACGCGAAGCTTAACGAGCAGTATCGCTTGGAGATTAACTCTGCGCTGAACCAAGAGGACCCAAGGACAGCTTGGGCGATGCTTCAAGGTATCAAGGCTGAACTAGATAAGGTCCAACCTGATGAGCAGATGACACCACAACGTGAGTGGCTAATCTCCGCACAGGAACAAGTCCAGAATCAGATGAACGCATGGACGAAAGCTCAAGCCAAGGCTCTGGACGATTCCATGAAGTCAATGAACa                                                                                                                                       >  1:214091/1‑301 (MQ=255)`  
                                                                                                                                                                      `ggTGAGGAACAGTGGAACGCTCTCATGGTCACAGCACAACGTTCTCAGTTTGAGACTGACGCGAAGCTGAACGAGCAGTATCGCTTGGAGATTAACTCTGCGCTGAACCAAGAGGACCCAAGGACAGCTTGGGCGATGCTTCAAGGTATCAAGGCTGAACTAGATAAGGTCCAACCTGATGAGCAGATGACACCACAACGTGAGTGGCTAATCTCCGCACAGGAACAAGTTCAGAATCAGATGAACGCATGGACGAAAGCTCAGGCCAAGGCTCTGGACGATTCCCTGAAGTCAATGAACa                                                                                                                                       >  1:272141/1‑301 (MQ=255)`  
                                                                                                                                                                            `gAACAGTGGAACGCTCTCATGGTCACAGCACAACGTTCTCAGTTTGAGACTGACGCGAAGCTGAACGAGCAGTATCGCTTGGAGATTAACTCTGCGCTGAACCAAGAGGACCCAAGGACAGCTTGGGCGATGCTTCAAGGTATCAAGGCTGAACTAGATAAGGTCCAACCTGATGAGCAGATGACACCACAACGTGAGTGTCTAATCTCCGCACAGGAACAAGTTCAGAATCAGATGAACGCATGGACGAAAGCTCAGGCCAAGGCTCTGGACGATTCCATGAAGTCAATGAACAAACTTg                                                                                                                                 >  1:388564/1‑301 (MQ=255)`  
                                                                                                                                                                              `aCAGTGGAACGCTCTCATGGTCACAGCACAACGTTCTCAGTTTGAGACTGACGCGAAGCTGAACGAGCAGTATCGCTTGGAGATTAACTCTGCGCTGAACCAAGAGGACCCAAGGACAGCTTGGGCGATGCTTCAAGGTATCAAGGCTGAACTAGATAAGGTCCAACCTGATGAGCAGATGACACCACAACGTGAGTGGCTAATCTCCGCACCGGAACAAGTTCAGAATCAGATGAACGCATGGACGAAAGCTCAGGCCAAGGCTCTGGACGATTCCATGAAGCCAATgctgtttctgata                                                                                                                               >  1:478888/1‑289 (MQ=255)`  
                                                                                                                                                                                         `ctctCATGGTCACAGCACAACGTTCTCAGTTTGAGACTGACGCGAAGCTGAACGAGCAGTATCGCTTGGAGATTAACTCTGCGCTGAACCAAGAGGACCCAAGGACAGCTTGGGCGATGCTTCAAGGTATCAAGGCTGAACTAGATAAGGTCCAACCTGATGAGCAGATGACACCACAACGTGAGTGGCTAATCTCCGCACAGGAACAAGTTCAGAATCAGATGAACGCATGGACGAAAGCTCAGGCCAAGGCTCTGGACGATTCCATGAAGTCAATGAACAAACTTGACGTAATCAAcct                                                                                                                    >  1:300765/1‑299 (MQ=255)`  
                                                                                                                                                                                         `ctctCATGGTCACAGCACAACGTTCTCAGTTTGAGACTGACGCGAAGCTGAACGAGCAGTATCGCTTGGAGATTAACTCTGCGCTGAACCAAGAGGACACAAGGACAGCTTGGGCGATGCTTCAAGGTATCAAGGCTGCACTAGATAAGGTCCAACCTGACGAGCAGATGACACCACAACGTGAGTGGCTAATCTCCGCACAGGAACAAGTTCAGAATCAGATGAACGCATGGACGAAAGCTCAGGCCAAGGCTCTGGACGATTCCATGAAGTCAATGAACAAACTTGACGTAATcaacaa                                                                                                                    >  1:373698/1‑301 (MQ=255)`  
                                                                                                                                                                                          `gctcATGGTCACAGCACAACGTTCTCAGTTTGATACTGACGCGAAGCTGAACGAGCAGTATCGCTTGGAGATTAACTCTGCGCTGAATCAAGAGGACCCAAGGACAGCTTGGGCGATGCTTCAAGGTATCAAGGCTGAACTAGATAAGGTCCAACCTGATGAGCAGATGACACCACAACGTGAGTGGCTAATCTCCGCACAGGAACAAGTTCAGAATCAGATGAACGCATGGACGAAAGCTCAGGCCAAGGCTCTGGACGATTCCATGAAGTCAATGAACAAACTTGACGTAATCAACAAg                                                                                                                   <  1:23065/300‑1 (MQ=255)`  
                                                                                                                                                                                           `ctcATGGTCACAGCACAACGTTCTCAGTTTGAGACTGACGCGAAGCTGAACGAGCAGTATCGCTTGGAGATTAACTCTGCGCTGAACCAAGAGGACCCAAGGACAGCTTGGGCGATGCTTCAAGGTATCAAGGCTGAACTAGATAAGGTCCAACCTGATGAGCAGATGACACCACAACGTGAGTGGCTAATCTCCGCACAGGAACAAGTTCAGAATCCGATGAACGCATGGACGAAAGCTCAGGCCAAGGCTCTGGACGATTCAATGAAGTCAATGAACAAACTTGACGGAATCAACAAga                                                                                                                  >  1:176500/1‑300 (MQ=255)`  
                                                                                                                                                                                             `cATGGTCACAGCACAACGTTCTCAGTTTGAGACTGACGCGAAGCTGAACGAGCAGTATCGCTTGGAGATTAACTCTGCGCTGAACCAAGAGGACCCAAGGACAGCTTGGGCGATGCTTCAAGGTATCAAGGCTGAACTAGATAAGGTCCAACCTGATGAGCAGATGACACCACAACGTGAGTGGCTAATCTCCGCACAGGAACAAGTTCAGAATCAGATGAACGCATGGACGAAAGCTCAGGCCAAGGCTCTGGACGATTCCATGAAGTCAATGAACAAACTTGACGTAATCAACAAGCaa                                                                                                                >  1:567430/1‑301 (MQ=255)`  
                                                                                                                                                                                                `ggTCACAGCACAACGTTCTCAGTTTGAGACTGACGCGAAGCTGAACGAGCAGTATCGCTTGGAGATTAACTCTGCGCTGAACCAAGAGGACCCAAGGACAGCTTGGGCGATGCTTCAAGGTATCAAGGCTGAACTAGCTAAGGTCCAACCTGATGAGCAGATGACACCACAACGTGAGTGGCTAATCTCCGCACAGGAACAAGTTCAGAATCAGATGAACGCATGGACGAAAGCTCAGGCCAAGGCTCTGGACGATTCCATGCAGTCAATGAACAAACTTGACGTAATCAACAAGCAATTc                                                                                                             >  1:328276/1‑301 (MQ=255)`  
                                                                                                                                                                                                 `gTCATAGCACAACGTTCTCAGTTTGAGACTGACGCGAAGCTGAACGAGCAGTATCGCTTGGAGATTAACTCTGCGCTGAACCAAGAGGACCCAAGGACAGCTTGGTCGATGCTTCAAGGTATCAAGGCTGAACTAGATAAGGTCCAACCTGATGAGCAGATGACACCACAACGTGAGTGGCTAATCTCCGCACAGGAACAAGTTCAGAATCAGATGAACGCATGGACGAAAGCTCAGGCCAAGGCTCTGGACGATTCCATGAAGTCAATGAACAAACTTGACGTAATCAACAAGCAATTcc                                                                                                            >  1:351523/1‑301 (MQ=255)`  
                                                                                                                                                                                                 `gTCACAGCACAACGTTCTCAGTTTGAGACTGACGCGAAGCTGAACGAGCAGTATCGCTTGGAGATTAACTCTGCGCTGAACCAAGAGGACCCAAGGACAGCTTGGGCGATGCTTCAAGGTATCAAGGCTGAACTAGATAAGGTCCAACCTGATGAGCAGATGACACCACAACGTGAGTGGCTAATCTCCGCCCAGGAACAAGTTCAGAATCAGATGAACGCATGGACGAAAGCTCAGGCCAAGGCTCTGGACGATTCCATGAAGTCAATGAACCAACTTGACgctgtctcttctacacatc                                                                                                            >  1:767986/1‑283 (MQ=255)`  
                                                                                                                                                                                                  `tCACAGCACAACGTTCTCAGTTTGAGACTGACGCGAAGCTGAACGAGCAGTATCGCTTGGAGATTAACTCTGCGCTGAACCAAGAGGACCCAAGGACAGCTTGGGCGATGCTTCAAGGTATCAAGGCTGAACTAGATAAGGTCCAACCTGATGAGCAGATGACACCACAACGTGAGTGGCTAATCTCCGCACAGGAACAAGTTCAGAATCAGATGAACGCATGGACGAAAGCTCAGGCCAAGGCTCTGGACGATTCCATGAAGTCAATGAACAAACTTGACGTAATCAACAAGCAATTCCa                                                                                                           >  1:416495/1‑301 (MQ=255)`  
                                                                                                                                                                                                     `caGCACAACGTTCTCAGTTTGAGACTGACGCGAAGCTGAACGAGCAGTATCGCTTGGAGATTAACTCTGCGCTGAACCAAGAGGACCCAAGGACAGCTTGGGCGATGCTTCAAGGTATCAAGGCTGAACTAGATAAGGTCCAACCTAATGAGCAGATGACACCACAACGTGAGTGGCTAATCTCCGCACAGGAACAAGTTCAGAATCAGATGAACGCATGGACGAAAGCTCAGGCCAAGGCTCTGGACGATTCCATGAAGTCAATGAACAAACTTGACgctgtctcttatacacatctccg                                                                                                        >  1:639384/1‑279 (MQ=255)`  
                                                                                                                                                                                                      `aGCACAACGTTCTCAGTTGGAGACTGAAGCGAAGCTGAACGATCAGTATCGCTTGGAGATTAACTCTGAGCTGAACCAAGAGGACCCAAGGACAGCTTGGGCGATGCTTCAAGGTATCAAGGCTGAACTAGATAAGGTCCAACCTGATGAGCAGATGACACCACAACGTGAGTGGCTAATCTCCGCACAGGAACAAGTTCAGAATCAGATGAACGCATGGACGAAAGCTCAGGCCAAGGCTCTGGACGATTCCATGAAGTCAATGAACAAACTTGACGTAATCAACAAGCAATTCCAGAAg                                                                                                       <  1:544134/301‑1 (MQ=255)`  
                                                                                                                                                                                                                 `ctcAGTTTGAGACGGACGAGAAGCGTAACGAGCAGTATCGCTTGGAGATTAAATCTGGGCTGAACCAAGAGGACCCAAGGAAAGCTTGGGCGATGCTTCAAGGTATCAAGGCTGAACTAGATAAGGTACAACCTGATGAGCAGATGACACCACAACGTGAGTGGCTAATCTCCGCACAGGAACAAGTTCAGAATCAGATGAACGCATGGACGAAAGCTCAGGCCAAGGCTCTGGGCGATTCCATGAAGTCAATGAACAAACTTGACGTAATCAACAAGCAATTCCAGAAGCGAATCAACgg                                                                                            <  1:107241/301‑1 (MQ=255)`  
                                                                                                                                                                                                                     `gTTTGATACTGACGCGAAGCTGAACGAGCAGTATCGCTTGGTGATTAACTCTGCGCTGAACCAAGAGGACCCAAGGACAGCTTGGGCGATGCTTCAAGGTATCAAGGCTGAACTAGATAAGGTCCAACCTGATGAGCAGATGACACCACAACGTGAGTGGCTAATCTCCGCACAGGAACAAGTTCAGAATCAGATGAACGCATGGACGAAAGCTCAGGCTAAGGCTCTGGACGATTCCATGAAGTCAATGAACAAACTTGACGTAATCAACAAGCAATTCCAGAAGCGAATCAACGGTGAg                                                                                        <  1:699748/301‑1 (MQ=255)`  
                                                                                                                                                                                                                     `gTTTGAGACTGACGCGAAGCTGAACGAGCAGTATCGCTTGGAGATTAACTCTGCGCTGAACCAAGAGGACCCAAGGACAGCTTGGGCGATGCTTCAAGGTATCAAGGCTGAACTAGATAAGGTCCAACCTGATGAGCAGATGACACCACAACGTGAGTGGCTAATCTCCGCACAGGAACAAGTTCAGAATCAGATGAACGCATGGACGAAAGCTCAGGCCAAGGCTCTGGACGATTCCATGAAGTCAATGAACAAACTTGACGTAATCAACAAGCAATTCCAGAAGCGAATCAACGGTGAg                                                                                        >  1:9324/1‑301 (MQ=255)`  
                                                                                                                                                                                                                      `tttGAGACTGACGCGAAGATGAACGAGCAGTATCGCTTGGAGATTAACTCTGCGCTGAACCAAGAGGACCCAAGGACAGCTTGGGCGATGCTTCAAGGTATCAAGGCTGAACTAGATAAGGTCCAACCTGATGAGCAGATGACACCACAACGTGAGTGGCTAATCTCCGCACAGGAACAAGTTCAGAATCAGATGAACGCATGGACGAAAGCTCAGGCCAAGGCTCTGGACGATTCCATGAAGTCAATGAACAAACTTGACGTAATCAACAAGCAATTCCAGAAGCGAATCAACGGTGAGt                                                                                       <  1:728056/301‑1 (MQ=255)`  
                                                                                                                                                                                                                                 `cgcgAAGCTGAACGAGCAGTATCGCTTGGAGATTAACTCTGCGCTGAACCAAGAGGACCCAAGGACAGCTTGGGCGATGCTTCAAGGTATCAAGGCTGAACTAGATAAGGTCCAACCTGATGAGCAGATGACACCACAACGTGAGTGGCTAATCTCCGAACAGGAACAAGTTCAGAATCAGATGAACGCATGGACGAAAGCTCAGGCCAAGGCTCTGGACGATTCCATGAAGTCAATGAACAAACTTGACGTAATCAACAAGCAATTCCAGAAGCGAATCAACGGTGAGTGGGTCTCAACg                                                                            <  1:789402/301‑1 (MQ=255)`  
                                                                                                                                                                                                                                     `aaGCTGAACGAGCAGTATCGCTTGGAGATTAACTCTGCGCTGAACCAAGAGGACCCAAGGACAGCTTGGGCGATGCTTCAAGGTATCAAGGCTGAACTAGATAAGGTCCAACCTGATGAGCAGATGACACCACAACGTGAGTGGCTAATCTCCGCACAGGAACAAGTTCAGAATCAGATGAACGCATGGACGAAAGCTCAGGCCAAGGCTCTGGACGATTCCATGAAGTCAATGAACAAACTTGACGTAATCAACAAGCAATTCCAGAAGCGAATCAACGGTGAGTGGGTCTCAACGGAtt                                                                        >  1:440430/1‑301 (MQ=255)`  
                                                                                                                                                                                                                                       `gCTGAACGAGCAGTATCGCTTGGAGATTAACTCTGCGCTGAACCAAGAGGACCCAAGGACAGCTTGGGCGATGCTTCAAGGTATCAAGGCTGAACTAGATAAGGTCCAACCTGATGAGCAGATGACACCACAACGTGAGTGGCTAATCTCCGCACAGGAACAAGTTCAGAATCAGATGAACGCATGGACGAAAGCTCAGGCCAAGGCTCTGGACGATTCCATGAAGTCAATGAACAAACTTGACGTAATCAACAAGCAATTCCAGAAGCGAATCAACGGTGAGTGGGTCTCAACGGAtttt                                                                      >  1:68391/1‑301 (MQ=255)`  
                                                                                                                                                                                                                                            `acaggCAGTATCGCTTGGAGATTAACTCTGCGCTGAACCAAGAGTACCCAAGGACAGCTTGGGCGATGCTTCAAGGTATCAAGGCTGAACTAGATAAGGTCCAACCTGATGAGCAGATGACACCACAACGTGAGTGGCTAATCTCCGCACAGGAACAAGTTCAGAATCAGATGAACGCATGGACGAAAGCTCAGGCCAAGGCTCTGGACGATTCCATGAAGTCAATGAACAAACTTGACGTAATCAACAAGCAATTCCAGAAGCGAATCAACGGTGAGTGGGTCTCAACGGATTTTAAGGa                                                                 <  1:115582/297‑1 (MQ=255)`  
                                                                                                                                                                                                                                                      `tCGCTTGGAGATTAACTCTGCGCTGAACCAAGAGGACCCAAGGACAGCTTGGGCGATGCTTCAAGGTATCAAGGCTGAACTAGATAAGGTCCAACCTGATGAGCAGATGACACCACAACGTGAGTGGCTAATCGCCGCACAGGAACAAGTTCAGAATCAGATGAACGCATGGACGAAAGCTCAGGCCAAGGCTCTGGACGATTCCATGAAGTCAATGAACAAACTTGACGTAATCAACAAGCAATTCCAGAAGCGAATCAACGGTGAGTGGGTCTCAACGGATTTTAAGGATATGCCAGTc                                                       <  1:198935/301‑1 (MQ=255)`  
                                                                                                                                                                                                                                                         `cTTGGAGATTAACTCTGCGCTGAACCAAGAGGACCCAAGGACAGCTTGGGCGATGCTTCAAGGTATCAAGGCTGAACTAGATAAGGTCCAACCTGATGAGCAGATGACACCACAACGTGAGTGGCTAATCTCCGCACAGGAACAAGTTCAGAATCAGATGAACGCATGGACGAAAGCTCAGGCCAAGGCTCTGGACGATTCCATGAAGTCAATGAACAAACTTGACGTAATCAACAAGCAATTCCAGAAGCGAATCAACGGTGAGTGGGTCTCAACGGATTTTAAGGactgtctcttatac                                                    >  1:508434/1‑288 (MQ=255)`  
                                                                                                                                                                                                                                                            `ggaGATTAACTCTGCGCTGAACCAAGAGGACCCAAGGACAGCTTGGGCGATGCTTCAAGGTATCAAGGCTGAACTAGATAAGGTCCAACCTGATGAGCAGATGACACCACAACGTGAGTGGCTAATCTCCGCACAGGAACAAGTTCAGAATCAGATGAACGCATGGACGAAAGCTCAGGCCAAGGCTCTGGACGATTCCATGAAGTCAATGAACAAACTTGACGTAATCAACAAGCAATTCCAGAAGCGAATCAACGGTGAGTGGGTCTCAACGGATTTTAAGGATATGCCAGTCAACgag                                                 <  1:617911/299‑1 (MQ=255)`  
                                                                                                                                                                                                                                                               `gATTAACTCTGCGCTGAACCAAGAGGACCCAAGGACATCTTGGGCGATGCTTCAAGGTATCAAGGCTGAACTAGATAAGGTCCAACCTGATGAGCAGATGACACCACAACGTGAGTGGCTAATCTCCGCACAGGAACAAGTTCAGAATCAGATGAACGCATGGACGAAAGCTCAGGCCAAGGCTCTGGACGATTCCATGAAGTCAATGAACAAACTTGACGTAATCAACAAGCAATTCCAGAAGCGAATCAACGGTGAGTGGGTCTCAACGGATTTTAAGGATATGccctgtctcttatac                                              >  1:486046/1‑288 (MQ=255)`  
                                                                                                                                                                                                                                                               `gATTAACTCTGCGCTGAACCAAGAGGACCCAAGGACAGCTTGGGCGATGCTTCAAGGTATCAAGGCTGAACTAGATAAGGTCCAACCTGATGAGCAGATGACACCACAACGTGAGTGGCTAATCTCCGCACAGGAACAAGTTCAGAATCAGATGAACGCATGGACGAAAGCTCAGGCCAAGGCTCTGGACGATTCCATGAAGTCAATGAACAAACTTGACGTAATCAACAAGCAATTCCAGAAGCGAATCAACGGTGAGTGGGTCTCAACGGATTTTAAGGATATGCCAGTCAACGAGAac                                              >  1:492505/1‑301 (MQ=255)`  
                                                                                                                                                                                                                                                               `gATTAACTCTGCGCTGAACCAAGAGGACCCAAGGACAGCTTGGGCGATGCTTCAAGGTATCAAGGCTGAACTAGATAAGGTCCAACCTGATGAGCAGATGACACCACAACGTGAGTGGCTAATCTCCGCACAGGAACAAGTTCAGAATCAGATGAACGCATGGACGAAAGCTCAGGCCAAGGCTCTGGACGATTCCATGAAGTCAATGAACAAACTTGACGTAATCAACAAGCAATTCCAGAAGCGAATCAACGGTGAGTGGGTCTCAACGGATTTTAAGGATATGCCAGTCAACGAGAac                                              >  1:137884/1‑301 (MQ=255)`  
                                                                                                                                                                                                                                                                  `tAACTCTGCGCTGAACCAAGAGGACCCAAGGACAGCTTGGGCGATGCTTCAAGGTATCAAGGCTGAACTAGATAAGGTCCAACCTGATGAGCAGATGACACCACAACGTGAGTGGCTAATCTCCGCACAGGAACAAGTTCAGAATCAGATGAACGCATGGACGAAAGCTCAGGCCAAGGCTCTGGACGATTCCATGAAGTCAATGAACAAACTTGACGTAATCAACAAGCAATTCCAGAAGCGAATCAACGGTGAGTGGGTCTCAACGGATTTTAAGGATATGCCAGTCAACGAGAACACt                                           >  1:13037/1‑301 (MQ=255)`  
                                                                                                                                                                                                                                                                           `gcTGAACCAAGAGGACCCAAGGACAGCTTGGGCGATGCTTCAAGGTATCAAGGCTGAACTAGATAAGGTCCAACCTGATGAGCAGATGACACCACAACGTGAGTGGCTAATCTCCGCACAGGAACAAGTTCAGAATCAGATGAACGCATGGACGAAAGCTCAGGCCAAGGCTCTGGACGATTCCATGAAGTCAATGAACAAACTTGACGTAATCAACAAGCAATTCCAGAAGCGAATCAACGGTGAGTGGGTCTCAACGGATTTTAAGGATATGCCAGTCAACGAGAACACTGGTGAGTTc                                  <  1:100022/301‑1 (MQ=255)`  
                                                                                                                                                                                                                                                                              `gAACCAAGAGGACCCAAGGACAGCTTGGGCGATGCTTCAAGGTATCAAGGCTGAACTAGATAAGGTCCAACCTGATGAGCAGATGACACCACAACGTGAGTGGCTAATCTCCGCACAGGAACAAGTTCAGAATCAGATGAACGCATGGACGAAAGCTCAGGCCAAGGCTCTGGACGATTCCATGAAGTCAATGAACAAACTTGACGTAATCAACAAGCAATTCCAGAAGCGAATCAACGGTGAGTGGGTCTCAACGGATTTTAAGGATATGCCAGTCAACGAGAACACTGGTGAGTTCAAg                               >  1:719861/1‑301 (MQ=255)`  
                                                                                                                                                                                                                                                                              `gAACCAAGAGGACCCAAGGACAGCTTGGGCGATGCTTCAAGGTATCAAGGCTGAACTAGATAAGGTCCAACCTGATGAGCAGATGACACCACAACGTGAGTGGCTAATCTCCGCACAGGAACAAGTTCAGAATCAGATGAACGCATGGACGAAAGCTCAGGCCAAGGCTCTGGACGATTCCATGAAGTCAATGAACAAACTTGACGTAATCAACAAGCAATTCCAGAAGCGAATCAACGGTGAGTGGGTCTCAACGGATGTTAAGGATATGCAAGTCAACGAGAACACTGGAGAGTTCAAg                               >  1:86555/1‑301 (MQ=255)`  
                                                                                                                                                                                                                                                                                `aCCAAGAGGACCCAAGGACAGCTTGGGCGATGCTTCAAGGTATCAAGGCTGAACTAGATAAGGTCCAACCTGATGAGCAGATGACACCACAACGTGAGTGGCTAATCTCCGCACAGGAACAAGTTCAGAATCAGATGAACGCATGGACGAAAGCTCAGGCCAAGGCTCTGGACGATTCCATGAAGTCAATGAACAAACTTGACGTAATCAACAAGCAATTCCAGAAGCGAATCAACGGTGAGTGGGTCTCAACGGATTTTAAGGATATGCCAGTCAACGAGAACACTGGTGAGTTCAAGCa                             >  1:742244/1‑301 (MQ=255)`  
                                                                                                                                                                                                                                                                                `aCCAAGAGGACCCAAGGACAGCTTGGGCGATGCTTCAAGGTATCAAGGCTGAACTAGATAAGGTCCAACCTGATGAGCAGATGACACCACAACGTGAGTGGCTAATCTCCGCACAGGAACAAGTTCAGAATCAGATGAACGCATGGACGAAAGCTCAGGCCAAGGCTCTGGACGATTCCATGAAGTCAATGAACAAACTTGACGTAATCAACAAGCAATTCCAGAAGCGAATCAACGGTGAGTGGGTCTCAACGGATTTTAAGGATATGCCAGTCAACGAGAACACTGGTGAGTTCAAGCa                             <  1:183138/301‑1 (MQ=255)`  
                                                                                                                                                                                                                                                                                `aCCAAGAGGACCCAAGGACAGCTTGGGCGATGCTTCAAGGTATCAAGGCTGAACTAGATAAGGTCCAACCTGATGAGCAGATGACACCACAACGTGAGTGGCTAATCTCCGCACAGGAACAAGTTCAGAATCAGATGAACGCATGGACGAAAGCTCAGGCCAAGGCTCTGGACGATTCCATGAAGTCAATGAACAAACTTGACGTAATCAACAAGCAATTCCAGAAGCGAATCAACGGTGAGTGGGTCTCAACGGATTTTAAGGATATGCCAGTCAACGAGAACACTGGTGAGTTCAAGCa                             <  1:664047/301‑1 (MQ=255)`  
                                                                                                                                                                                                                                                                                         `aCCCAAGGACAGCTTGGGCGATGCTTCAAGGTATCAAGGCTGAACTAGATAAGGTCCAACCTGATGAGCAGATGACACCACAACGTGAGTGGCTAATCTCCGCACAGGAACAAGTTCAGAATCAGATGAACGCATGGACGAAAGCTCAGGCCAAGGCTCTGGACGATTCCATGAAGTCAATGAACAAACTTGACGTAATCAACAAGCAATTCCAGAAGCGAATCAACGGTGAGTGGGTCTCAACGGATTTTAAGGATATGCCAGTCAACGAGAACACTGGTGAGTTCAAGCATAGCGatat                    <  1:471780/301‑1 (MQ=255)`  
                                                                                                                                                                                                                                                                                          `cccAAGGACAGCTTGGGCGATGCTTCAAGGTATCAAGGCTGAACTAGATAAGGTCCAACCTGATGAGCAGATGACACCACAACGTGAGTGGCTAATCTCCGCACAGGAACAAGTTCAGAATCAGATGAACGCATGGACGAAAGCTCAGGCCAAGGCTCTGGACGATTCCATGAAGTCAATGAACAAACTTGACGTAATCAACAAGCAATTCCAGAAGCGAATCAACGGTGAGTGGGTCTCAACGGATTTTAAGGATATGCCAGTCAACGAGAACACTGGTGAGTTCAAGCactgtctctta                   >  1:447489/1‑291 (MQ=255)`  
                                                                                                                                                                                                                                                                                              `aGGACAGCTTGGGCGATGCTTCAAGGTATCAAGGCTGAACTAGATAAGGTCCAACCTGATGAGCAGATGACACCACAACGTGAGTGGCTAATCTCCGCACAGGAACAAGTTCAGAATCAGATGAACGCATGGACGAAAGCTCCGGCCCAGGCTCTGGACGATTCCATGAAGTCAATGAACAAACTTGACGTAATCAACAAGCAATTCCAGAAGCGAATCAACGGTGAGTGGGTCTCAACGGATTTTAAGGATATGCCAGTCAACTAGAACACTGGTGAGTTCAAGCATAGCGATATGGTTa               >  1:394726/1‑301 (MQ=255)`  
                                                                                                                                                                                                                                                                                              `aGGACAGCTTGGGCGATGCTTCAAGGTATCAAGGCTGAACTAGATAAGGTCCAACCTGATGAACAGATGACACCACAACGTGAGTGGCTAATCTCCGAACAAGAACAAGTTCAGAATCAGATGAACGCATGGACGAAAGCTCAGGCCAAGGCTCTGGACGATTCCATGAAGTCAATGAACAAACTTGACGTAATCAACAAGCAATTCCAGAAGCGAATCAACGGTGAGTGGGTCTCAACGGATTTTAAGGATATGCCAGTCAACGAGAACACTGGTGAGTTCAAGCATAGCGATATGGTTa               <  1:582496/301‑1 (MQ=255)`  
                                                                                                                                                                                                                                                                                               `ggACAGCGTGGGCGATGCTTCAAGGTATCAAGGCTGAACTAGAAAAGGTCCAACCTGATGAGCAGATGACACCACAACGTGAGTGGCTAATCTCCGCACAGGAACAAGTTCAGAATCAGATGAACGCATGGACGAAAGCTCAGGCCAAGGCTCTGGACGATTCCATGAAGTCAATGAACAAACTTGACGTAATCAACAAGCAATTCCAGAAGCGAATCAACGGTGAGTGGGTCTCAACGGATTTTAAGGATATGCCAGTCAACGAGAACACTGGTGAGTTCAAGCATAGCGATATGGATaa              >  1:320066/1‑301 (MQ=255)`  
                                                                                                                                                                                                                                                                                                `gtataagagacagaTGCTGCAAGGTAGCAAGGCTGAACTAGATAAGGTCCAACCTGATGAGCAGATGACACCACAACGTGAGTGGCTAATCTCCGCACAGGAACAAGTTCAGAATCAGATGAACGCATGGACGAAAGCTCAGGCCAAGGCTCTGGACGATTCCATGAAGTCAATGAACAAACTTGACGTAATCAACAAGCAATTCCAGAAGCGAATCAACGGTGAGTGGGTCTCAACGGATTTTAAGGATATGCCAGTCAACGAGAACACTGGTGAGTTCAAGCATAGCGATATGGTTAAc             <  1:239191/290‑1 (MQ=255)`  
                                                                                                                                                                                                                                                                                                `gACAGCTTGGGCGATGCTTCAAGGTATCAAGGATGAACTAGATAAGGTCCAACCTGATGAGCAGATGACACCACAACGTGAGTGGCTAATCTCCGCACAGGAACAAGTTCAGAATCAGATGAACGCATGGACGAAAGCTCAGGCCAAGGCTCTGGACGATTCCATGAAGTCAATGAACAAACTTGACGTAATCAACAAGCAATTCCAGAAGCGAATCAACGGTGAGTGGGTCTCAACGGATTTTAAGGATATGCCAGTCAACGAGAACACTGGTGAGTTCAAGCATAGCGATATGGTTAAc             <  1:515587/301‑1 (MQ=255)`  
                                                                                                                                                                                                                                                                                                  `cAGCTTGGGCGATGCTTCAAGGTATCAAGGCTGAACTAGATAAGGTCCAACCTGATGAGCAGATGACACCACAACGTGAGTGGCTAATCTCCGCACAGGAACAAGTTCAGAATCAGATGAACGCATGGACGAAAGCTCAGGCCAAGGCTCTGGACGATTCCATGAAGTCAATGAACAAACTTGACGTAATCAACAAGCAATTCCAGAAGCGAATCAACGGTGAGTGGGTCTCAACGGATTTTAAGGATATGCCAGTCAACGAGAACACTGGTGAGTTCAAGCATAGCGATATGGTTAAcct           >  1:455086/1‑299 (MQ=255)`  
                                                                                                                                                                                                                                                                                                  `aggCTTGGGCGATGCTTCAAGGTATCAAGGCTGAACTAGATAAGGTCCAACCTGATGAGCAGATGACACCACAACGTGAGTGGCTAATCTCCGCACAGGAACAAGTTCAGAATCAGATGAACGCATGGACGAAAGCTCAGGCCAAGGCTCTGGACGATTCCATGAAGTCAATGAACAAACTTGACGTAATCAACAAGCAATTCCAGAAGCGAATCAACGGTGAGTGGGTCTCAACGGATTTTAAGGATATGCCAGTCAACGAGAACACTGGTGAGTTCAAGCATAGCGATATGGTTAACTa           <  1:796466/299‑1 (MQ=255)`  
                                                                                                                                                                                                                                                                                                    `gCTTGGGCGATGCTTCAAGGTATCAAGGCTGAACTAGATAAGGTCCAACCTGATGAGCAGATGACACCACAACGTGAGTGGCTAATCTCCGCACAGGAACAAGTTCAGAATCAGATGAACGCATGGACGAAAGCTCAGGCCAAGGCTCTGGACGATTCCATGAAGTCAATGAACAAACTTGACGTAATCAACAAGCAATTCCAGAAGCGAATCAACGGTGAGTGGGTCTCAACGGATTTTAAGGATATGCCAGTCAACGAGAACACTGGTGAGTTCAAGCATAGCGATATggctttctctt         >  1:801914/1‑292 (MQ=255)`  
                                                                                                                                                                                                                                                                                                    `gCTTGGGCGATGCTTCAAGGTATCAAGGCTGAACTAGATAAGGTCCAACCTGATGAGCAGATGACACCACAACGTGAGTGGCTAATCTCCGCACAGGAACAAGTTCAGAATCAGATGAACGCATGGACGAAAGCTCAGGCCAAGGCTCTGGACGATTCCATGAAGTCAATGAACAAACTTGACGTAATCAACAAGCAATTCCAGAAGCGAATCAACGGTGAGTGGGTCTCAACGGATTTTAAGGATATGCCAGTCAACGAGAACACTGGTGAGTTCAAGCATAGCGATATGGTTAACTAcc         >  1:464702/1‑300 (MQ=255)`  

                                                                                                                                                                                                                                                                                                           `|`                                                                                                                                                                                                                                                                                                              
`CGGTCTGGTTACTGGCGCAATCCCATCTGATGCTCAAGCCACACAGCTTATAAGCCAAGCGTTCAGTGACGCTTCTAGCCGTGCTGGTGGTGCTGACTTCCTGATGCGAGTCGGTGACAAGAAGGTAACACTTAACGGAGCCACTACGACTTACCGAGAGTTGATTGGTGAGGAACAGTGGAACGCTCTCATGGTCACAGCACAACGTTCTCAGTTTGAGACTGACGCGAAGCTGAACGAGCAGTATCGCTTGAAGATTAACTCTGCGCTGAACCAAGAGGACCCAAGGACAGCTTGGGAGATGCTTCAAGGTATCAAGGCTGAACTAGATAAGGTCCAACCTGATGAGCAGATGACACCACAACGTGAGTGGCTAATCTCCGCACAGGAACAAGTTCAGAATCAGATGAACGCATGGACGAAAGCTCAGGCCAAGGCTCTGGACGATTCCATGAAGTCAATGAACAAACTTGACGTAATCGACAAGCAATTCCAGAAGCGAATCAACGGTGAGTGGGTCTCAACGGATTTTAAGGATATGCCAGTCAACGAGAACACTGGTGAGTTCAAGCATAGCGATATGGTTAACTACGCCAATAA  >  NC_001604.1/29005‑29604`

  

Alignment Legend

Aligned base mismatch/match (shaded by quality score): `ATCG/ATCG` `< 3 ≤` `ATCG/ATCG` `< 15 ≤` `ATCG/ATCG` `< 27 ≤` `ATCG/ATCG` `< 37 ≤` `ATCG/ATCG` `< 38 ≤` `ATCG/ATCG`

Unaligned base: `atcg`    Masked matching base: `atcg`    Alignment gap: `‑`    Deleted base: `‑`
